# Molecular Basis of IgE-Mediated Shrimp Allergy and Heat Desensitization

**DOI:** 10.3390/nu13103397

**Published:** 2021-09-27

**Authors:** PeiAo Zhang, Jihui Gao, Huilian Che, Wentong Xue, Dong Yang

**Affiliations:** 1 College of Food Science & Nutritional Engineering, China Agricultural University, Beijing 100083, China; 2018306100617@cau.edu.cn (P.Z.); jhgao@cau.edu.cn (J.G.); chehuilian@cau.edu.cn (H.C.); xwt@cau.edu.cn (W.X.); 2 Beijing Key Laboratory of Functional Food from Plant Resources, College of Food Science & Nutritional Engineering, China Agricultural University, Beijing 100083, China

**Keywords:** Chinese shrimp, tropomyosin, variable heavy chain, IgE, allergy

## Abstract

Crustacean allergy, especially to shrimp, is the most predominant cause of seafood allergy. However, due to the high flexibility of immunoglobulin E (IgE), its three-dimensional structure remains unsolved, and the molecular mechanism of shrimp allergen recognition is unknown. Here a chimeric IgE was built in silico, and its variable region in the light chain was replaced with sequences derived from shrimp tropomyosin (TM)-allergic patients. A variety of allergenic peptides from the Chinese shrimp TM were built, treated with heating, and subjected to IgE binding in silico. Amino acid analysis shows that the amino acid residue conservation in shrimp TM contributes to eliciting an IgE-mediated immune response. In the shrimp-allergic IgE, Glu98 in the light chain and other critical residues that recognize allergens from shrimp are implicated in the molecular basis of IgE-mediated shrimp allergy. Heat treatment could alter the conformations of TM allergenic peptides, impact their intramolecular hydrogen bonding, and subsequently decrease the binding between these peptides and IgE. We found Glu98 as the characteristic amino acid residue in the light chain of IgE to recognize general shrimp-allergic sequences, and heat-induced conformational change generally desensitizes shrimp allergens.

## 1. Introduction

Seafood, especially fish and shellfish, are becoming more and more consumed since they are a source of valuable protein contents for the general population [1]. On the other hand, crustaceans are suggested to be the main elicitors of food allergy worldwide [2]. Shrimp is the most predominant cause of seafood allergy and has been extensively studied among crustaceans, and it is most frequently associated with immunoglobulin E (IgE)-mediated type I hypersensitive reactions among food allergy patients [3]. Previous studies have identified tropomyosin (TM) as the major allergens of crustaceans, while new targets such as troponin C, myosin light chain, sarcoplasmic Ca^2+^-binding protein, triosephosphate isomerase, hemocyanin, filamin C, and pyruvate kinase as allergenic proteins are also emerging [4,5,6,7,8,9,10]. IgE antibody recognition is the characteristic response in a typical shrimp-allergic individual, and it could be used as a diagnosis tool for shrimp allergy detection [11].

Different treatments have been applied to alleviate the allergenicity of shrimp. For example, extracts from boiled shrimp exhibited lower IgE binding than extracts from raw shrimp, indicating heat treatment on the allergenic proteins could be an efficient way to reduce shrimp allergenicity [12,13]. Frying, marination, and fermentation also decreased the recognition of shrimp extracts on human IgE [13]. High-intensity ultrasounds oxidized the cysteine, methionine, and lysine residues in TM and degraded TM by induced free radicals, consequently altering the secondary structures of TM and decreasing its allergenicity [14]. Microwave treatment at 125 °C strongly modified the secondary structure of shrimp proteins, and induced a 75% reduction in shrimp allergenicity, indicating the significance of secondary structures in holding allergenicity [15]. The probiotic *Bifidobacterium infantis* 14.518 alleviates TM-induced allergy by inducing tolerogenic dendritic cell maturation and gut microbiota adjustment in some of the population [16]. It was recently shown that *Lactobacillus Casei* Zhang alleviates the TM-associated allergenicity by switching the antibody isotopes to the tolerogenic pattern, and other bacteria in the gut microbiota also play important roles in modulating our immune system [17,18]. Intradermal administration of DNA vaccine pMED171 exhibited down-regulation of the allergic response to TM in murine models [19]. Treatment of sensitized murine models with Chitosan oligosaccharide significantly reduces serum IgE levels [20]. The effort to reduce shrimp allergy is unceasingly going on while there is no study on the molecular basis of IgE-mediated shrimp allergenicity.

Heat treatment, in various forms including steaming, boiling, baking, and frying, is a commonly used method to process food for the purpose of enhancing texture, flavor, and microbial safety. Meanwhile, the allergenicity of food allergens may decrease or increase during heat treatment [21]. The aforementioned microwave treatment reaches its maximum reduction in TM allergenicity when combined with heat treatment at 125 °C [15]. Nonetheless, how heat treatment impacts the conformational epitopes and their subsequent recognition by IgE remains unknown.

Maillard reaction takes place during dry-heat cooking, and covalent attachment via the carbonyl group of reducing sugars to the ε-amino group of lysine residue in TM reduces its allergenicity up to 60% [22]. On the other hand, artificial covalent modification of TM by transglutaminase and tyrosinase reduces the IgE-induction capacity of TM [23]. These studies all suggest the significance of these key amino acid residues in eliciting IgE-mediated immunogenicity. Previously, the key amino acids eliciting allergenicity in the Chinese Shrimp (*Penaeus chinensis*) have been identified; however, the detailed molecular mechanism of how these mutations are immunologically significant remains unknown [24].

In this work, we study the effect of heat treatment on the conformational changes of the major allergenic proteins, TM, in the Chinese Shrimp (*Penaeus chinensis*) via computational simulation. The conformations of TM under their native state (25 °C) and boiling temperature (100 °C) were simulated, and critical mutations affecting their allergenicity were analyzed in parallel. The allergenicity of TM epitopes with critical mutations, native and after heat treatment, was assessed by simulation of their binding to human IgE, derived from shrimp-allergic patients in silico. 

## 2. Materials and Methods

### 2.1. Preparation of the Shrimp-Allergic IgE Structure

The three-dimensional structure of IgE allergic to the Chinese shrimp was obtained by replacing the amino acid residues in the variable region of the light chain of an X-ray diffraction structure (IgE-β-lactoglobulin complex, PDB: 2R56) with amino acid sequences from the corresponding variable region of the IgE light chain from the only previously reported Chinese Shrimp TM-allergic human subject (DIQMTQSPSSLSASVGDTVTITCRASQDINGYLAWYQQKPGKVPKLLIYGHLLCNQGSRLGSAAVDLGQISLSPSAACSLKMLQLITAKSIPLPLGRSAKGPRWKS) [25]. The chimeric structure was then subjected to molecular dynamic (MD) simulations, and is described as follows. The BIOVIA Discovery Studio (DS) software V16.1.0 (Dassault Systèmes, San Diego, CA, USA) was employed to perform the MD simulations based on the CHARMm36 molecular mechanics and molecular dynamics force field engine. In the DS standard dynamics cascade, all systems were solvated in a spherical container with a radius of 36.7 Å using the explicit periodic boundary water model and neutralized by the addition of sodium cation and chloride anion to an ionic concentration of 0.2. Initially, the system underwent two energy minimization steps: 1000 steps of the steepest descent minimization and 2000 steps of conjugate gradient minimization with the adopted Newton–Raphson algorithm. Three steps of heating, equilibration, and production were performed afterward as follows. The whole system was heated from 50 K to 310.15 K in 4 ps without constraints, and then the equilibrium step was run at 310.15 K for 20 ps without constraints. The following production step was run at 310.15 K and a pressure of 1.0 for 200 ps with typed NPT and no constraints. The electrostatic parameter was set to automatic, which recognizes the periodic environment and uses the Particle Mesh Ewald (PME) electrostatic calculation. Among the 100 conformations generated, the one with the lowest total energy was selected for the following study [26].

### 2.2. Preparation and Heat Treatment of the Allergenic Peptides

The initial structure of the allergenic peptides, including 9 TM peptides and 4 mutants on critical amino acid residues, were generated with PyMol (Schrödinger, New York, NY, USA). MD simulation was carried out to obtain the conformations of all above peptides at native and heat-treated conditions with modifications on the temperatures. For native peptide conformation, the temperature at heating, equilibration, and production steps was set at 298.15 K, and for heat-treated peptide conformation, the above temperatures were set at 373.15 K.

### 2.3. Assessment of the Allergenicity of Heat-Treated Peptides

The allergenicity of heat-treated peptides, including those with mutations on their critical amino acid residues, was tested by examining their binding to the above-mentioned chimeric IgE. Molecular docking between different peptides and the chimeric IgE was performed with the DS CDOCKER module. Both the peptides and IgE were prepared at pH 7.5 and ionic strength of 0.2, and the variable regions of IgE were selected as the binding site with the radius set at 0.5 Å. Over 1000 dynamic steps and 10 random conformations were generated for the initial ligand conformation at a temperature of 1000 K, accompanied with 10 orientations. The following simulated annealing was performed with 2000 steps, heating to a target temperature of 700 K, and 5000 steps, cooling to a target temperature of 300 K. Among the 10 peptide-IgE complexes generated, the complex with the lowest CDOCKER energy was subjected to interaction analysis and calculation of binding energy with the calculate binding energies module equipped in the DS. LigPlot (EMBL-EBI Groups, Cambridgeshire, UK) was used to analyze the hydrophobic interactions and PyMol was used to analyze the hydrogen bonding.

## 3. Results

### 3.1. Sequence Analysis of TM Epitope Peptides

The Chinese shrimp TM epitope peptide sequences studied here are from previous wet lab experiments (Table 1) [24]. There is a cross-reactivity among crustacean TMs and those from other invertebrates such as mollusks and cockroaches [27,28]. For crustacean TMs, those from brown shrimp, sand shrimp, black tiger prawn, American lobster, spiny lobster, oyster, and red crab have been identified along with their amino acid sequences [29,30,31,32,33,34,35,36]. The T3 sequence from the Chinese shrimp (QKRMQQLENDLDQV) is identical with that of brown shrimp, black tiger prawn, kuruma prawn, and sand shrimp, but slightly different from that in pink shrimp [37].

**Figure 1:**
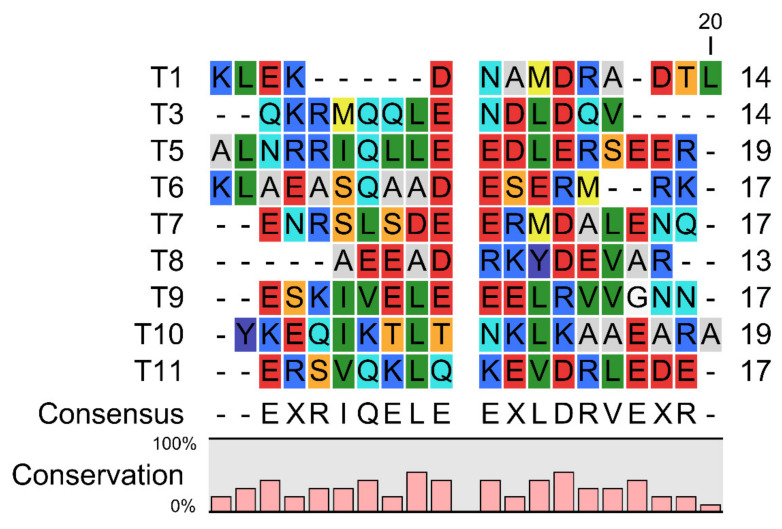
Sequence alignment of the Chinese Shrimp TM allergenic peptides involved in this study. Red indicates amino acid residues D, E; yellow indicates M; blue indicates R, K; violet indicates Y; dark green indicates I, L, V; cyan indicates Q, N; light grey indicates A; orange indicates S, T; white indicates G.

Here we aligned these TM epitope peptides, and found that there is a conservation of Glu (E), Arg (R), Ile(I), Gln (Q), Leu (L), and Asp (D) residues in these peptides (Figure 1). This indicates a potential role of these amino acid residues involved in eliciting the immunogenicity mediated by IgE.

### 3.2. Heat Treatment on the Conformational Changes of TM Peptides

All TM allergenic peptides exhibited considerate conformational changes compared to their native states (25 °C) with root mean square deviation (RMSD) ranging from 2.190 Å to 3.448 Å (Table 2). Heat treatment rendered a solvent accessible surface area (SASA) reduction in T3, T7, T8, T10, and T11 but an exposure of more SASA in T1, T5, T6, and T9. Among them, SASA of T10 decreased most significantly by 106.5 Å^2^ after heat treatment. The distance between the α-carbon of the N- and C-terminals was measured and compared as a rough indicator of how protein conformation deviated from its native form. Most of the TM epitope peptides curled up except for T3 and T9, whose terminal distances increased by ~1.5 Å. Most of the TM epitope peptides exhibited increased intramolecular hydrogen bonding after heat treatment except for T1, T5, T8 and T9, whose intramolecular hydrogen bonding did not change.

### 3.3. IgE Interaction with Native and Heat-Treated TM Peptides

**Figure 2:**
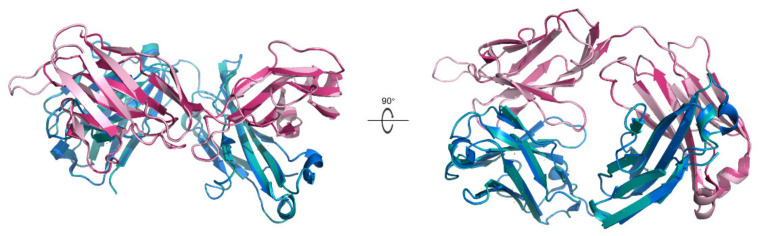
Alignment of the simulated chimeric IgE structure with the X-ray diffraction one. The simulated chimeric IgE structure (light chain in light pink and heavy chain in teal) is aligned with the X-ray diffraction structure of IgE (light chain in hot pink and heavy chain in marine) in the β-lactoglobulin-IgE complex (PDB ID:2R56). Left is the side view and right is one rotated 90° along the x-axis.

Here a chimeric IgE that contains the variable region recognizing shrimp allergenic TM epitopes in the light chain of the Fab region was constructed (Figure 2). There is an extra loop in the chimeric IgE, which is from the replacement of the variable region in the Fab light chain with the identified amino acid sequence from shrimp-allergic patients. Thus, this chimeric variable region can theoretically recognize the Chinese Shrimp TM epitopes. Besides that, the chimeric IgE structure is very similar to that of the IgE in the IgE-β-lactoglobulin complex with an RMSD value of only 0.122 Å. Thus, the subsequent immunogenicity study of the Chinese Shrimp allergenic peptides was performed with this chimeric IgE.

**Figure 3:**
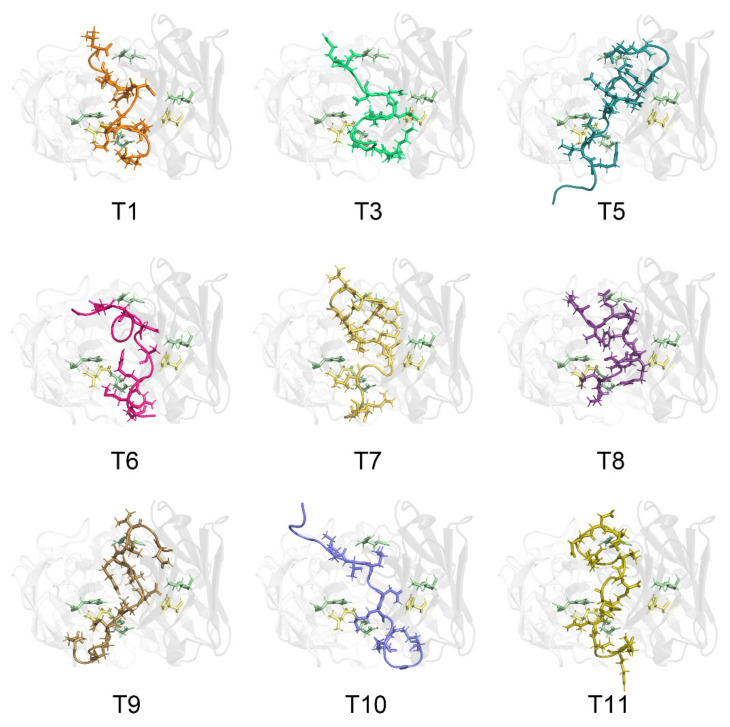
Detailed interaction between the Chinese Shrimp allergenic peptides and the chimeric IgE. The IgE light chain is colored in grey90 and the heavy chain is in grey40. For a clear view, the transparency of the IgE structure is set as 80%. Amino acid residues His59 in IgE heavy chain and Leu97 in IgE light chain are shown in sticks and colored in pale yellow. Glu98, His31 in IgE light chain, Arg101, Lys57 in IgE heavy chain involved in both hydrogen bonding and hydrophobic interactions are shown in sticks and colored in pale green. The epitope peptides are shown in cartoon and key amino acid residues involved in the interaction with IgE are shown in sticks and colored as orange (T1), lime green (T3), deep teal (T5), hot pink (T6), yellow orange (T7), violet purple (T8), sand (T9), slate (T10) and olive (T11).

All the native TM allergenic peptides were recognized by chimeric IgE with various degrees of binding affinities (Figure 3), as suggested by different binding energies between them, ranging from −2.89 kcal/mol to −30.49 kcal/mol (Table 3). The TM peptides generally adjusted their structural conformation upon binding to IgE with RMSD values ranging from 4.10 Å to 9.03 Å. For most of the TM allergenic peptides, their SASA decreased by a range of −69.17 Å^2^ to −229.70 Å^2^ upon interaction with the complementary-determining regions.

Heat treatment indeed altered the interaction between allergenic peptides and IgE (Table 3). The majority binding energies of TM allergenic peptides increased after heat treatment except peptides T3, T6 and T10, indicating that heat treatment could possibly decrease the allergenicity of TM proteins. For TM peptides, heat-treated ones generated less structural adjustment upon binding with IgE compared with their native forms, except T5 and T10.

Generally, Glu98 in the light chain and Arg101 in the heavy chain formed hydrogen bonds with all TM allergenic peptides. Lys57 in the heavy chain formed hydrogen bonds with all TM allergenic peptides except T1, and His31 in the light chain formed hydrogen bonds with all the peptides except T6, T9, T10 and T11. These four amino acid residues play critical roles in IgE-recognizing allergens via hydrogen bonding. Glu98 and His31 in the light chain and Lys57 and Arg101 in the heavy chain form hydrophobic interactions with all the TM allergenic peptides. Leu97 in the light chain formed hydrophobic interactions with all peptides except T10. Tyr37 in the light chain formed hydrophobic interactions with all peptides except T8. Thr99 and Asn33 in the light chain formed hydrophobic interactions with all peptides except T5. His59 in the heavy chain formed hydrophobic interactions with all peptides except T9 and T11. Thr105 in the heavy chain formed hydrophobic interactions with all peptides except T6 and T9. These ten amino acid residues play critical roles in IgE recognition of shrimp allergens via hydrophobic interactions.

### 3.4. Critical Amino Acid Residues Involved in IgE Recognition of TM Allergenic Peptides

As identified in previous studies, there are some critical TM amino acid residues involved in the recognition of these TM epitope peptides by IgE [24]. Generally, amino acid residues Arg, Glu, Asp, and Lys in TM form the most hydrogen bonds with IgE residues and amino acid residues Arg, Glu, Leu, Lys, and Asp in TM form most hydrophobic interactions with IgE. These amino acid residues are characteristic residues in shrimp allergenic proteins.

Here we also performed in silico mutations on some of these allergenic peptides and examined their interaction with IgE (Table 4). For T7, mutations of E131, R140, D142, and Q147 brought about structural deviations from the wild-type with RMSD values ranging from 2.283 Å to 3.641 Å. All the mutations on T7 peptide led to a more frizzy structure, as suggested by the decreased terminal distances of these mutations. Meanwhile, the peptide structures were more organized as there were more intramolecular hydrogen bonds formed in these mutations. These mutations indeed affected the peptides’ interaction with IgE, as indicated by the increased binding energies. On the one hand, E131 and R140 mutations led to less SASA buried (up to 231.39 Å^2^) upon interaction with IgE while D142 and Q147 led to SASA exposure compared to the wild-type (as to 101.02 Å^2^).

## 4. Discussion

It is reported that patients with shrimp allergy may also suffer from ingestions of other crustaceans such as lobster, crab, prawn, and crayfish, suggesting a significant similarity of the IgE-binding epitopes in these allergens [38]. Among shrimps, TM allergenic peptide sequences could even be identical as previously mentioned. For different Chinese shrimp TM-linear epitopes, there is a conservation of Glu, Arg, Ile, Gln, Leu, and Asp in their amino acid sequences. Here, computational study demonstrated that these conserved amino acid residues are characteristic allergenicity-inducing residues. Arg, Glu, Asp, and Lys are recognized by IgE via hydrogen bonding while Arg, Glu, Leu, Lys, and Asp are recognized by IgE via hydrophobic interactions. Our results are consistent with previous studies of 4-hydroxy-2-noneal treatment which found that Leu and Lys of shrimp TM are the key amino acid residues recognized by IgE [39]. The results are also consistent with previous studies of peptide library screening, which found that Glu, Arg, Ile, Gln, and Asp are the critical residues involved in TM immunogenicity [40].

As for residue in the antibody, critical amino acid residues Glu98 and His31 in the light chain and Arg101 and Lys57 in the heavy chain recognize the allergenic peptides via hydrogen bonding. Glu98, Leu97, His31, Tyr37, Thr99, and Asn33 in the light chain, and Lys57 and Arg101 in the heavy chain recognize the peptides via hydrophobic interactions. Moreover, these amino acid residues are the molecular basis of shrimp allergen recognition by IgE. Currently, the majority of studies are focused on identifying the epitope sequences of TM and other allergens while there is no study on the IgE amino acid residues involved in shrimp TM recognition. Thus, to the best of our knowledge, this study offers the first insights into the structural basis of immune responses to shrimp allergenic proteins.

Although invertebrate TMs are more stable to heat than non-allergenic vertebrate TMs, heat treatment in general did alter the conformational changes of shrimp TM allergenic peptides in all aspects in this study [41]. This is consistent with experimental evidence that boiling reduces shrimp allergenicity [12]. Here, different peptides exhibited different structural changes upon heat treatment. One study suggested that the lack of α-helix folding might cause immunogenicity of invertebrate TMs [42]. Other studies indicated that reduction in shrimp TM allergenicity rose from the increase in β-sheets and decrease in turns [15,22]. In this study, most of the TM peptides formed new hydrogen bonds after heat treatment, suggesting an alteration of the secondary structure of Chinese shrimp TM under heat treatment. It is worth mentioning that as our study focuses on the fragments of TM allergenic peptides, the result could be different when examining these sequences when they are part of the intact TM proteins, or even when these proteins are part of the shrimp tissue rather than purified proteins. For example, boiling also lowered the IgE binding to shrimp extracts and enhanced IgE binding to the purified TM from boiled shrimp [12]. Nevertheless, the in silico study here obtained similar results to that of *in vivo* experiments, explained the critical amino acid residues involved in IgE interaction with TM allergenic peptides, and thus offered a molecular view of thermal treatment on the shrimp allergens [24].

## 5. Conclusions

In conclusion, there is a conservation of Glu, Arg, Ile, Gln, Leu, and Asp in the major shrimp allergenic TM. Among them, Arg, Glu, Asp, and Lys interact with IgE via hydrogen bonding while Arg, Glu, Leu, Lys, and Asp interact with IgE via hydrophobic interactions. In the shrimp allergic IgE, Glu98, and His31 in the light chain and Arg101 and Lys57 in the heavy chain recognize the allergens via hydrogen bonding while Glu98, Leu97, His31, Tyr37, Thr99, and Asn33 in the light chain and Lys57 and Arg101 in the heavy chain recognize the allergens via hydrophobic interactions. Heat treatment could alter the conformations of TM allergenic peptides, impact their intramolecular hydrogen bonding, and decrease the binding between these peptides and IgE. Our results not only revealed the molecular basis of IgE-mediated shrimp TM allergy but also the mechanism of heat-treated desensitization of TM epitope peptides.

## Figures and Tables

**Table 1:** Allergenic peptides studied in this research.

Peptide ^1^	Sequence	Position ^2^	Mutation ^3^
T1	KLEKDNAMDRADTL	12−25	
T3	QKRMQQLENDLDQV	47−60	
T5	ALNRRIQLLEEDLERSEER	87−105	
T6	KLAEASQAADESERMRK	112−128	
T7	ENRSLSDEERMDALENQ	131−147	
T7-E131	ANRSLSDEERMDALENQ		E131
T7-R140	ENRSLSDEEAMDALENQ		R140
T7-D142	ENRSLSDEERMAALENQ		D142
T7-Q147	ENRSLSDEERMDALENA		Q147
T8	AEEADRKYDEVAR	155−167	
T9	ESKIVELEEELRVVGNN	187−203	
T10	YKEQIKTLTNKLKAAEARA	221−239	
T11	ERSVQKLQKEVDRLEDE	243−259	

^1^ Peptide is the exact sequence of part of the allergenic protein. ^2^ Position indicates the location of these peptides in the original allergenic protein. ^3^ Mutations indicate these amino acid residues mutated into alanine.

**Table 2:** Conformational change of heat-treated TM peptides.

Peptide	RMSD (Å) ^1^	ΔSASA (Å^2^) ^2^	Terminal Distance (Å) ^3^		Number of H-Bonds ^4^	
			Native	Heated	Native	Heated
T1	2.469	11.40	38.0	31.2	5	5
T3	3.376	−1.57	37.7	39.1	2	5
T5	2.542	4.79	57.1	54.9	4	4
T6	2.680	37.02	49.9	49.5	1	4
T7	3.278	−149.64	40.6	36.6	1	3
T8	2.880	−29.47	34.1	33.0	2	2
T9	2.570	35.04	44.6	46.1	1	1
T10	3.448	−106.49	55.3	51.2	0	2
T11	2.190	−10.65	45.7	38.3	8	11

^1^ RMSD is the root mean square deviation of the heat-treated peptide compared to its native conformation. ^2^ ΔSASA is the solvent accessible surface area change of the heat-treated peptide compared to its native conformation. ^3^ Terminal distance is the distance between the C_α_ of the N-terminal and the C-terminal of the peptide at each condition. ^4^ Number of H-hydrogen bonds is the number of intra-molecular hydrogen bonds maintaining the peptide at this condition.

**Table 3:** Parameters between IgE interaction with native and heat-treated TM peptides.

Peptide	Number of H-Bonds ^1^		Number of Hydrophobic Interactions ^2^		Binding Energy (kcal/mol)		RMSD (Å) ^3^		ΔSASA (Å^2^) ^4^	
	Native	Heated	Native	Heated	Native	Heated	Native	Heated	Native	Heated
T1	14	5	64	35	−17.28	−7.83	6.62	5.61	−171.37	−255.80
T3	12	6	58	64	−12.16	−21.11	5.47	3.69	−209.82	−176.00
T5	8	9	52	73	−19.01	−18.21	6.11	7.27	−89.79	−313.73
T6	9	6	46	46	−2.89	−12.53	9.03	5.62	−82.85	−0.50
T7	12	11	62	72	−24.16	−2.56	5.06	4.45	−165.80	−170.14
T8	8	5	59	42	−10.19	−0.084	7.38	6.63	−89.50	−77.24
T9	4	5	54	59	−4.52	−1.53	5.72	5.15	−121.52	−119.43
T10	7	15	42	85	−10.08	−20.27	4.10	5.75	−229.70	−189.70
T11	8	4	62	47	−30.49	−3.10	7.23	3.17	−69.17	−87.17

^1^ Number of H-bonds is the number of inter-molecular hydrogen bonds between the peptide and IgE. ^2^ Number of Hydrophobic Interactions is the number of inter-molecular hydrophobic interactions between the peptide and IgE. ^3^ RMSD is the epitope structural change brought by binding of peptide to IgE. ^4^ ΔSASA is the epitope solvent accessible surface area change in its IgE-bound form compared to its apo- conformation.

**Table 4:** Effects of mutations on the IgE interaction with native and heated TM peptides.

Peptide	RMSD ^1^ (Å)	Terminal Distance ^2^ (Å)	Number of H-Bonds ^3^	Binding Energy (kcal/mol)	ΔSASA (Å^2^) ^4^
T7-WT	-	57.1	1	−24.16	−165.80
T7-E131	2.283	41.5	7	−12.49	−228.30
T7-R140	3.641	41.1	7	−12.84	−231.39
T7-D142	3.294	44.9	11	−14.05	−108.21
T7-Q147	3.303	37.9	13	−17.91	−101.02

^1^ This RMSD indicates the structural change brought about by the mutation. ^2^ Terminal distance is the distance between the Cα of the N-terminal and the C-terminal of the peptide at each condition. ^3^ Number of H-bonds is the number of intra-molecular hydrogen bonds maintained in the peptide at this condition. ^4^ ΔSASA is the solvent accessible surface area change of the peptide in its IgE-bound form compared to its apo- conformation.

## Data Availability

Data are contained within the article.
